# Dual Trajectories of Social Isolation and Dementia in Older Adults: A Population-Based Longitudinal Study

**DOI:** 10.1093/geroni/igaa057.1022

**Published:** 2020-12-16

**Authors:** Xiaoling Xiang, Patrick Ho Lam Lai, Yihang Sun, Ruth Dunkle

**Affiliations:** 1 University of Michigan, Ann Arbor, Michigan, United States; 2 University of Michigan-Ann Arbor, Ypsilanti, Michigan, United States

## Abstract

The purpose of this study was to identify patterns of changes in social isolation and dementia and the interrelations between these developmental trajectories. The study sample included 7,609 Medicare beneficiaries age 65 and older from the National Health and Aging Trends Study 2011 through 2018 surveys. A group-based dual trajectory modeling approach was used to identify distinct groups of developmental trajectories for social isolation and dementia status over the 8-year period. The dual model provided estimates of conditional and joint probabilities linking the two sets of trajectory groups. Changes in social isolation over an 8-year period followed four trajectories: rarely isolated (62.2%), steady increase (13.5%), steady decrease (7.4%), and persistently isolated (16.9%). Changes in dementia risk also followed four trajectories: persistently low risk (80.4%), increasing with early onset (3.9%), increasing with late onset (4.5%), and persistently high risk (11.2%). Over two-third (68%) of the persistently low dementia group were also in the rarely isolated group. Both increasing dementia groups were composed mainly of individuals from the increasing social isolation group (40-43%) and persistently isolated group (24-29%). The persistently high dementia group had the most overlap with the decreasing social isolation group (47%), followed by the persistently isolated group (28%). For the most part, social isolation and dementia evolve in the same direction for older adults over an 8-year period. However, the pattern of associations between these developmental trajectories is complex and may be reversed among long-term dementia survivors.

